# A Novel Method for Interferometric Phase Estimation in Dual-Channel Cancellation

**DOI:** 10.3390/s22239356

**Published:** 2022-12-01

**Authors:** Long Huang, Aifang Liu, Zuzhen Huang, Hui Xu, Dong Han

**Affiliations:** Nanjing Research Institute of Electronic Technology, Nanjing 210039, China

**Keywords:** SAR anti-jamming methods, SAR jamming suppression, dual-channel cancellation, cosine similarity

## Abstract

Multichannel SAR systems have grown rapidly over the past decade due to their powerful high-resolution and wide-swath (HRWS) capabilities. Because spatially separated channels also have the potential to suppress jamming, dual-channel cancellation is a general method that is effective regardless of the type of jamming signal. In this paper, the principle of dual-channel cancellation (DCC) is introduced, and several practical problems using DCC are also discussed. Moreover, this paper emphasizes interferometric phase estimation, which is the key to DCC. If the jamming-to-signal ratio (JSR) is high, the interferometric phase can be estimated accurately from the interferometry of two channel signals, but estimation becomes rather difficult when the JSR decreases. To solve the problem of interferometric phase estimation under a low JSR, a novel interferometric phase estimation method using cosine similarity is proposed in this paper. L-band airborne dual-channel SAR is performed to investigate the applicability of the method. The results not only prove that cosine similarity is an effective method for interferometric phase estimation, but also demonstrate the potential of DCC in the SAR anti-jamming processing.

## 1. Introduction

Multichannel synthetic aperture radar (SAR) allows continuous observation of the Earth’s surface regardless of weather and daylight, which has motivated the rapid development of SAR technology. Among the different methods, ground moving target indication is used to monitor the velocities of moving targets [1,2,3,4,5,6], while interferometric SAR (InSAR) generates digital elevation maps of the ground surface [7,8,9].

SAR enjoys great benefits in the face of electronic counter measures due to its coherent processing and wide bandwidth. However, if the jamming power is high enough, SAR performance can still be seriously affected. In most instances, the jamming signal is similar and may even be identical to the radar signal. It is difficult to distinguish the interfering signal from the radar echo because they overlap in both the time and frequency domains [1,10,11].

Several anti-jamming methods have been proposed, such as the RFI suppression method [12], WBI suppression method [13], STAP method [14], spatial location feature recognition method [15], and other methods [16,17,18,19]. Evaluation methods for the anti-jamming performance of SAR have also been studied [20,21]. Taking full advantage of multichannel approaches, several researchers have proposed multichannel SAR methods [14,22,23,24,25,26,27,28,29], but they are usually limited to specific jamming types. To deal with this problem, Ma [30] proposed a general method, dual-channel cancellation, which is effective regardless of the jamming type when dealing with an active jammer. Theoretical analysis and simulations have demonstrated the effectiveness of DCC, but problems have arisen in practice. Ma pointed out that without an accurate estimate of the interferometric phase of the radiation source (IPSR), the jamming signal will not be completely cancelled [30].

In this paper, other practical problems that may be faced using DCC are discussed, such as the estimation of the IPSR, the effect of co-registration of two images on DCC, and resolution degradation after DCC. Among these problems, the most intractable one is the estimation of IPSR.

Although DCC has been proven to be effective in simulations [28,30,31], no flight experiment has ever been reported. To validate the DCC method and demonstrate the above issues, an experiment was conducted using an L-band airborne dual-channel SAR sensor. We used the DCC method and compared the SAR images before and after DCC, and the results of the experiment were quite satisfactory.

The remainder of this paper is organized as follows. In Section 2, the signal model of the dual channel cancellation is introduced. Combined with simulation, the estimation of IPSR based on cosine similarity is then studied. After that, the effects of co-registration, resolution degradation, and dark straps are analyzed. In Section 3, the airborne experimental results at the L-band are shown to prove the validity and practicality of our method. Finally, we discuss our findings and draw conclusions in Section 4 and Section 5.

## 2. Materials and Methods

For dual-channel SAR, the complex images that are processed are highly correlated, regardless of whether the two channels are along-track or cross-track. Interferometric images of the two channels contain certain information about the height and velocity of the imaging area, and the two intensity images are almost identical if the amplitude-phase errors are neglected or compensated for. On the other hand, the jamming signals that the two channels receive have a small time lag and a corresponding phase shift, which change slowly according to the geometrical relationship between the SAR channels and the source of radiation. If the IPSR is estimated precisely, then the jamming signals can be eliminated by phase compensation and cancellation.

### 2.1. Signal Model of the Dual Channel Cancellation

From the study in [32], for an arbitrary point target, we assume that the echoes of channel 1 and channel 2 received during the slow time *η* are written as follows:

(1) $$s_{\mathrm{pt}1}\left(t\right)=A_{\mathrm{pt}}\left(t\right)\exp\left[j\phi_{\mathrm{pt}}\left(t\right)\right]$$

(2) $$s_{\mathrm{pt}2}\left(t\right)=A_{\mathrm{pt}}\left(t-\Delta t_{\mathrm{pt}}\right)\exp\left[j\phi_{\mathrm{pt}}\left(t\right)+j\Delta \phi_{\mathrm{pt}}\left(\eta \right)\right]$$

where t is quick time, $A_{\mathrm{pt}}\left(t\right)$ and $\phi_{\mathrm{pt}}\left(t\right)$ are the amplitude and phase of the echo that channel 1 received, $\Delta t_{\mathrm{pt}}$ is the time lag of the echoes between the two channels, and $\Delta \phi_{\mathrm{pt}}\left(\eta \right)$ is the phase shift of the echoes between the two channels.

Let a source of radiation be deployed in the imaging area, transmitting signals of the same frequency band toward the dual-channel SAR. The jamming signals received by channel 1 and channel 2 can be written as

(3) $$s_{j1}(t)=A_{j}(t)\exp[j\phi_{j}(t)]$$

(4) $$s_{j2}(t)=A_{j}(t-\Delta t_{j})\exp[j\phi_{j}(t)+j\Delta \phi_{j}(\eta )]$$

where $A_{j}\left(t\right)$ and $\phi_{j}\left(t\right)$ are the amplitude and phase of the jamming signals, respectively, and $\Delta \phi_{j}\left(t\right)$ is the IPSR. Note that $\Delta \phi_{j}\left(\eta \right)$ is the slow time variant. Usually, if the two channels are not too far away, the time lag $\Delta t_{j}$ is neglected, and $A_{j}\left(t-\Delta t_{j}\right)$ in (4) becomes $A_{j}\left(t\right)$. The signals that the two channels receive are the summation of the ground echoes and the jamming signals, which are

(5) $$s_{1}(t)=A_{\mathrm{pt}}(t)\exp[j\phi_{\mathrm{pt}}(t)]+A_{j}(t)\exp[j\phi_{j}(t)]$$

(6) $$s_{2}(t)=A_{\mathrm{pt}}(t)\exp[j\phi_{\mathrm{pt}}(t)+j\Delta \phi_{\mathrm{pt}}(\eta )]+A_{j}(t)\exp[j\phi_{j}(t)+j\Delta \phi_{j}(\eta )]$$

Compensate (5) with $\exp\left[\Delta \phi_{j}\left(\eta \right)\right]$, and form the difference with (6). This results in the following difference signal:

(7) $$\begin{array}{ll} s_{d}(t) & =s_{2}(t)-s_{1}(t)\exp[j\Delta \phi_{j}(\eta )]\\ & =A_{\mathrm{pt}}(t)\exp[j\phi_{\mathrm{pt}}(t)+j\Delta \phi_{\mathrm{pt}}(\eta )]-A_{\mathrm{pt}}(t)\exp[j\phi_{\mathrm{pt}}(t)+j\Delta \phi_{j}(\eta )] \end{array}$$

From (7), we can see that the jamming signals are eliminated. Meanwhile, the phase of the echoes is affected by $\Delta \phi_{j}\left(\eta \right)$, wherein if $\Delta \phi_{\mathrm{pt}}\left(\eta \right)$ is close to $\Delta \phi_{j}\left(\eta \right)$, and $s_{d}\left(t\right)$ is close to 0, it means that some of the echoes would also be cancelled.

### 2.2. Estimation of the Interferometric Phase of the Source of Radiation

The most fundamental step of DCC is the estimation of the IPSR, which is denoted as $\Delta \phi_{j}(\eta )$ in the equations above.

The interferometric phase of the dual channels (IPDC) is the phase difference of the vector addition of the ground echoes and the jamming signals.

On the one hand, if the jamming-to-signal ratio (JSR) is high enough, the jamming signals dominate the echoes, and IPSR dominates the interferometric phase, which means that IPSR is very close to IPDC. Since IPDC is easy to compute, in this case, IPSR is considered equal to IPDC [30]. The cost of this approximation is that IPSR is not precise and will cause residual interference after DCC.

**Figure 1:**
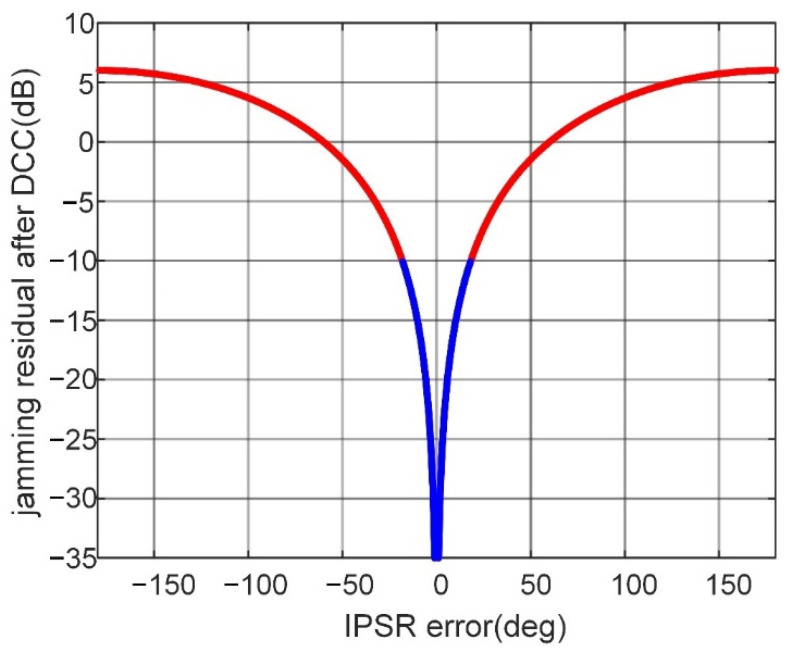
Jamming residual with IPSR error.

The jamming residuals after DCC with different IPSR errors are obtained by simulation, as shown in Figure 1. From Figure 1, we can see that the IPSR error decreases with the reduction of the jamming residual after DCC. Generally, cancellation with residual interference less than −10 dB can be considered effective. In such a case, the IPSR error should not be greater than 18°, which corresponds to the blue curve in Figure 1.

On the other hand, if the JSR is not high enough, the estimation of IPSR will become rather intractable. The difference between IPSR and IPDC shows relatively strong ground echoes.

**Figure 2:**
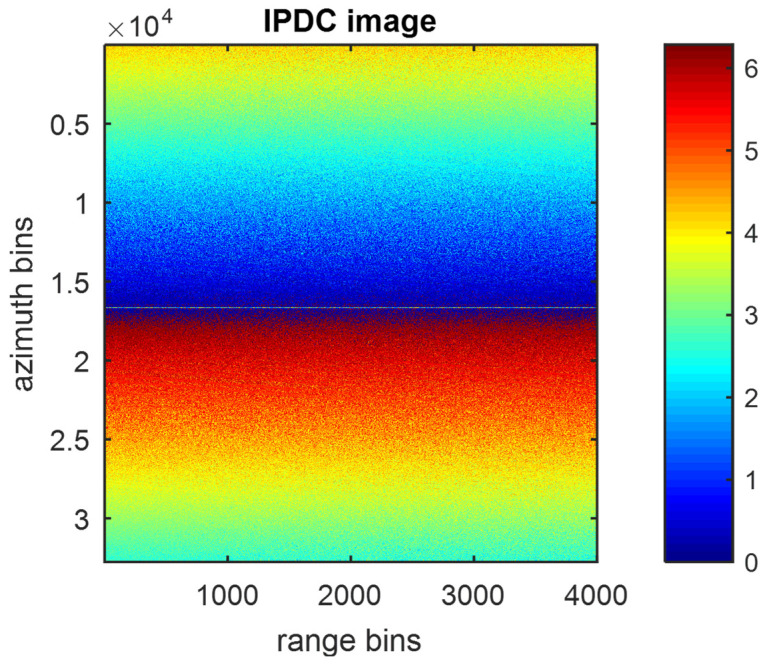
IPDC image of 10 dB JSR.

**Figure 3:**
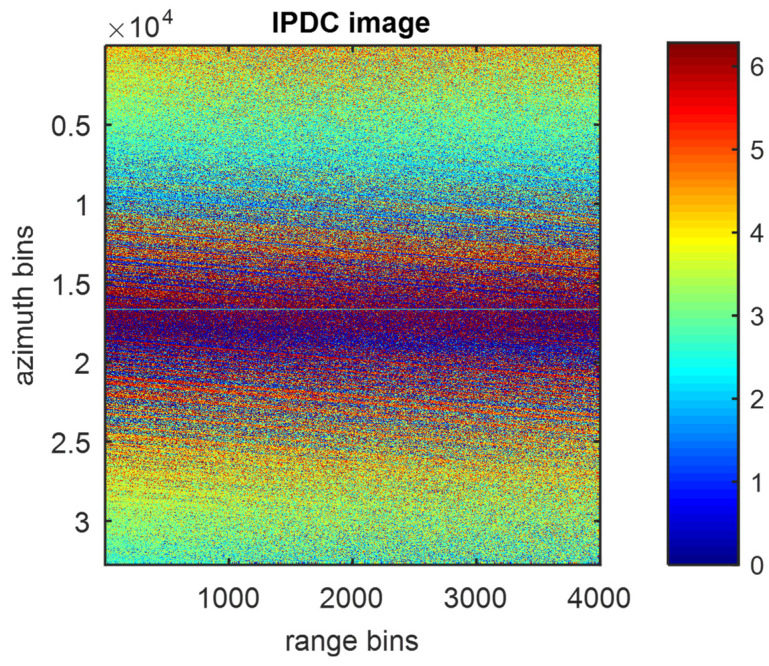
IPDC image of 0 dB JSR.

Figure 2 and Figure 3 show the IPDC under different JSRs, in which the color map represents the interferometric phase ranging from 0 to 2π. It is clear that a higher JSR can result in a smoother IPDC. If one examines the arbitrary azimuth bin in Figure 2, it can be found that the IPDCs along the range bins are nearly the same, which is consistent with the previous analysis. In addition, the distribution of IPDCs along the azimuth direction can be easily identified as being nearly linear.

However, the performance of IPDC will be seriously affected by the value of the JSR. As is clearly shown in Figure 3, when the JSR is reduced to 0 dB, the IPDC image will behave more noisily than in Figure 2, in which the IPDC distribution pattern along the azimuth direction can barely be identified.

**Figure 4:**
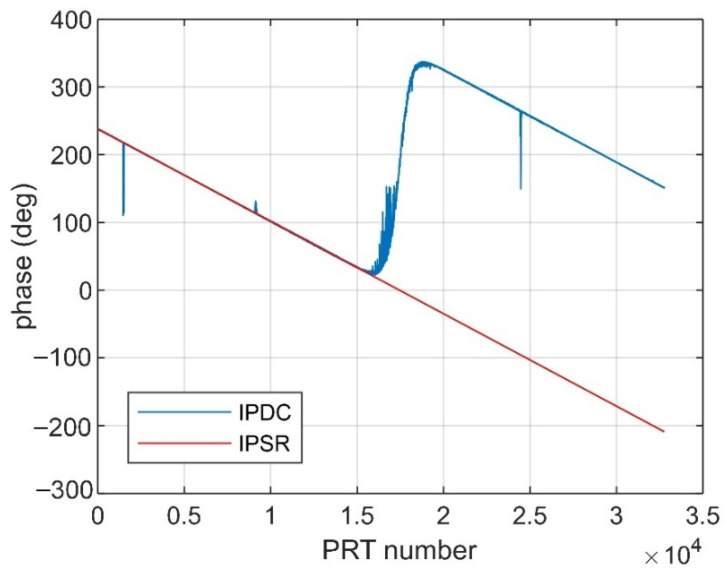
IPSRs and IPDCs along azimuth (10 dB JSR).

**Figure 5:**
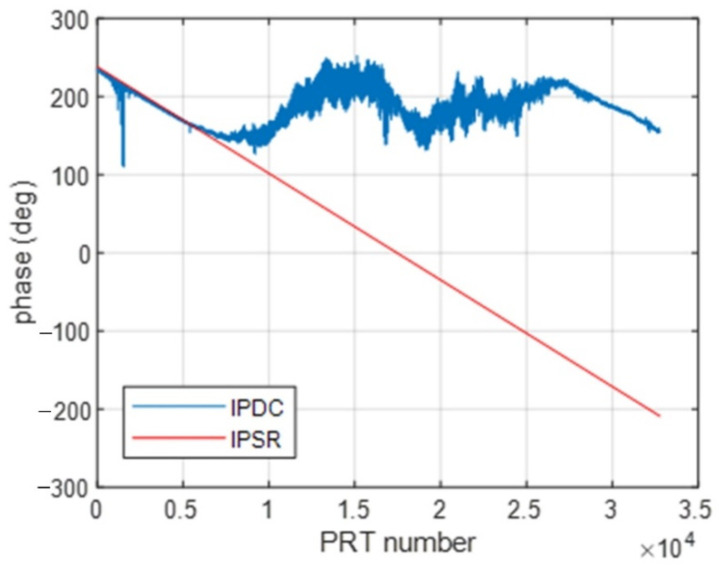
IPSRs and IPDCs along azimuth (0 dB JSR).

Figure 4 and Figure 5 show the IPSR and IPDC distribution comparison along the azimuth direction under different JSRs.

As is shown in Figure 4, when the JSR is 10 dB, there is a good match between the IPSR and IPDC, in which we can note that the IPDC is wrapped from 0° to 360°. However, in Figure 5, where the JSR is 0 dB, the IPSRs are quite different from the IPDCs and will be clearly not effective in DCC.

To address this problem, a traversal algorithm using cosine similarity is presented. For an arbitrary pulse repeat time (PRT), we traverse the IPSR from 0 to 2π and perform DCC. As the interference signals are different from the SAR echoes in certain aspects (for example, in the frequency domain), then we can use the similarity of the spectrum of pure SAR echoes and that of DCC signals to determine the optimal IPSR estimation.

In this method, cosine similarity is used to describe the similarity in the spectrum shapes, which is calculated as below:

(8) $$\gamma \left(IPSR\right)=\frac{\sum_{k=1}^{n}F_{1k}F_{2k}\left(IPSR\right)}{\sqrt{\sum_{k=1}^{n}F_{1k}^{2}}\sqrt{\sum_{k=1}^{n}F_{2k}^{2}\left(IPSR\right)}}$$

where $F_{1k}$ demotes the spectrum of the pure SAR echoes, $F_{2k}\left(IPSR\right)$ denotes the spectrum of the DCC signal corresponding to IPSR, and γ(IPSR) is the cosine similarity corresponding to IPSR. When the IPSR meets the optimal value, the cosine similarity will reach its peak.

Since the IPSR changes slowly along the azimuth direction, one does not need to perform the traversal algorithm for each PRT. To simplify the calculation, one can repeat this process along the azimuth direction at a suitable PRT interval and use interpolation to achieve IPSR of each PRT.

**Figure 6:**
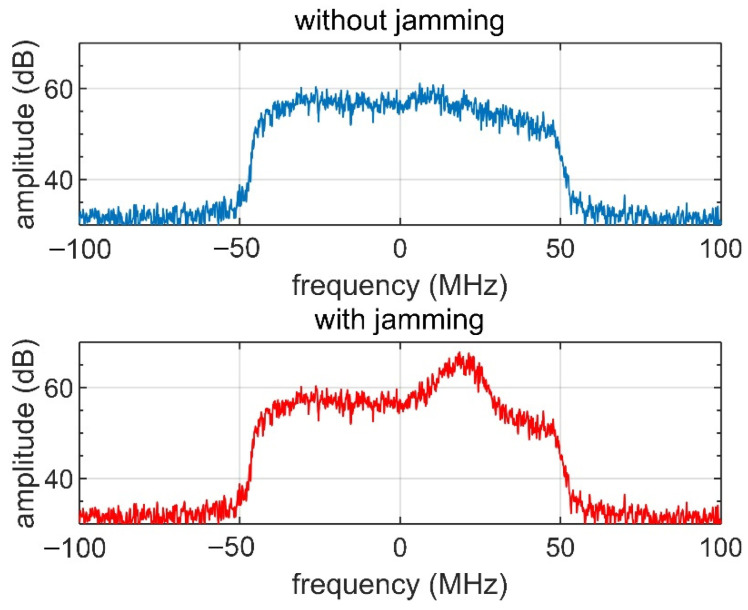
Spectrum without jamming and with 0 dB jamming.

To verify this method, a preliminary simulation based on real data is conducted. The data were obtained using an L-band airborne SAR, whose main parameters are listed in Table 1. The original data were achieved without jamming, and the spectrum is shown in the upper side of Figure 6. In the simulation, a jamming signal with 30 MHz bandwidth is added to the original data, in which the JSR is 0 dB. The shape of the spectrum is then distorted, as shown on the lower side of Figure 6. After the jamming operation, we traverse the IPSR from 0 to 2π, perform DCC, and examine the shape of the spectrum until a close shape is matched.

**Figure 7:**
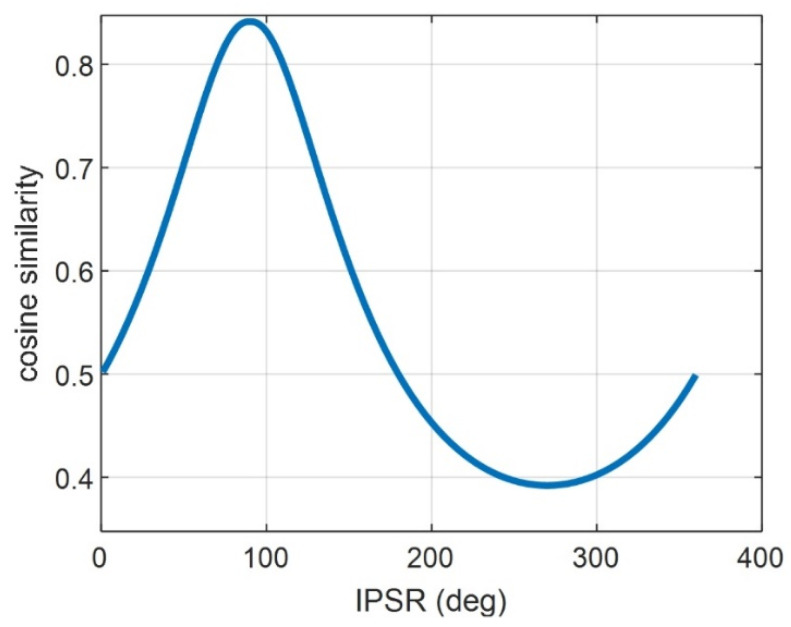
Cosine similarity with IPSR.

In this simulation, the ideal IPSR is set to 90°, and the IPSR interval is set to 1°. Under this situation, the cosine similarity of different IPSR values could be obtained, as clearly shown in Figure 7. As we can see from the Figure 7, the cosine similarity will reach the peak value of 0.8414 when the IPSR is 90°, which is consistent with the simulation condition.

**Figure 8:**
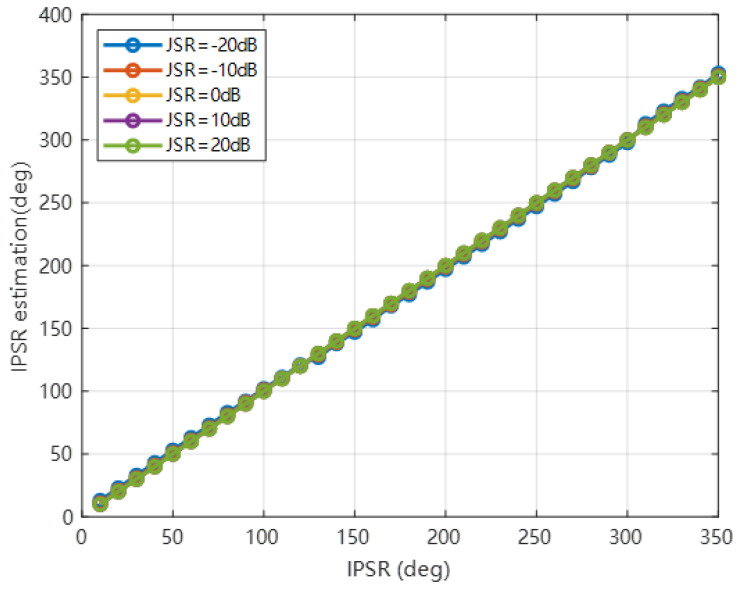
IPSR estimation under different JSRs.

To verify the validity of the cosine similarity method in different situations, we perform IPSR estimation simulations under different JSR, as shown in Figure 8. In Figure 8, it is demonstrated that the IPSR estimation results are quite accurate under different IPSR angles. Moreover, we can find that the cosine similarity method can also achieve good performance, even at −10 dB and −20 dB for different JSRs.

### 2.3. Effects of Co-Registration

In Section 2.1, the time lag between two SAR channels is neglected, which is true for the along-track DCC, since the distance between the two channels is usually less than 1 m, and such an along-track distance would cause a time lag that is much less than that of a sample interval. However, for cross-track DCC, the channel distance is usually in meters for airborne platforms and can even reach hundreds of meters for spaceborne platforms. The two channels are distributed in the cross-track direction, which makes it possible to cause a time lag of tens to thousands of sample intervals. Thus, range co-registration for interference signals will be needed for cross-track DCC.

Assuming that IPSR estimation is precise, the jamming residual after DCC suffers from a co-registration error. The residual is dependent on the jamming bandwidth to signal bandwidth ratio since the narrowband signal phase changes slower than that of the wideband signal.

**Figure 9:**
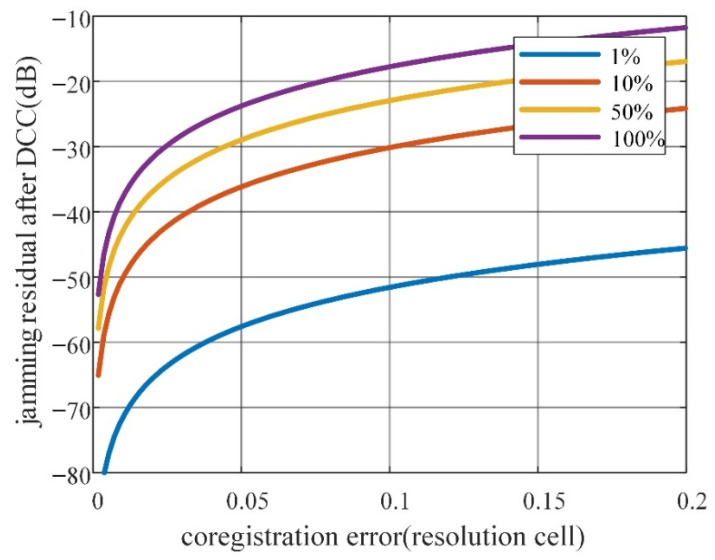
Jamming residual with co-registration error of different jamming bandwidth to signal bandwidth ratio.

Figure 9 shows the jamming residual with the co-registration error of different jamming bandwidth to signal bandwidth ratios. The curves show that the jamming residual suffers from the jamming bandwidth to signal bandwidth ratio and co-registration error, which will grow with either the jamming bandwidth to signal bandwidth ratio or the co-registration error. As we can see from Figure 9, when the jamming bandwidth is equal to the signal bandwidth, a 0.2 resolution cell co-registration error will approximately cause −12 dB jamming residual, which is commonly considered to be acceptable.

### 2.4. Ghost Images

**Figure 10:**
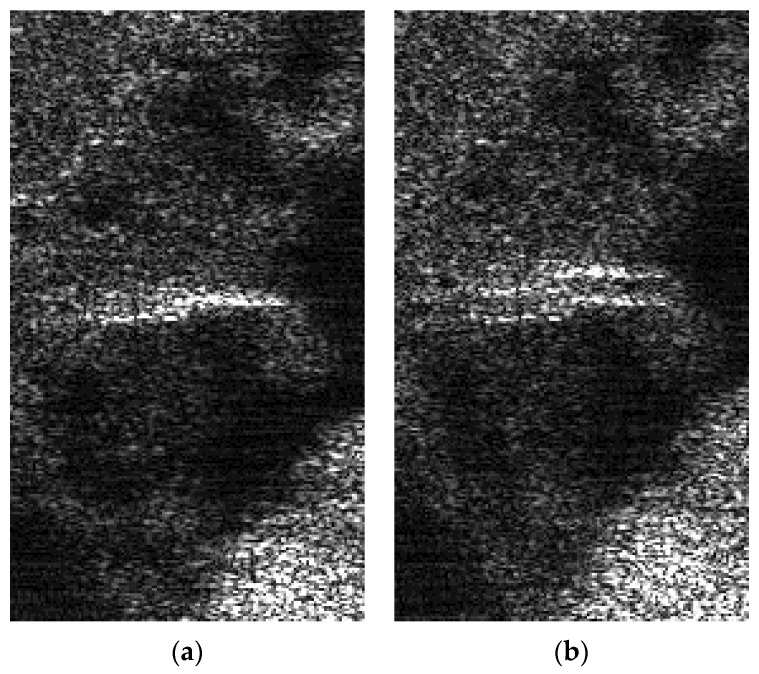
Demonstration of azimuth resolution degradation. (**a**) SAR image before DCC. (**b**) SAR image after DCC.

Along-track DCC usually leads to ghost images. There is a co-registration step in along-track multichannel SAR processing. However, since the jamming signals must be processed in the same PRT, there will be no azimuth co-registration step, which causes ghost images. The degree of the ghost image is proportional to the channel distance since the channel distance of a multichannel SAR is typically close to its azimuth resolution. Figure 10 gives a simulation demonstration that shows how, if the channel distance is much too large, then clear ghost images would appear.

### 2.5. Dark Strap

There is usually more than one dark strap on DCC images. The direction of the dark straps mainly depends on the channel configuration. In cross-track DCC, the interferometric phase map is periodic along the range direction. Those pixels whose interferometric phase is close to IPSR are cancelled, forming dark straps along the azimuth direction and cycle along the range direction. In along-track DCC, however, the dark straps are located along the range direction and cycle along the azimuth direction.

The dark strap is the negative effect of DCC, which will slightly affect the interpretation of SAR images. The period of the dark straps is inversely proportional to the channel distance [22]. Ma proposed a three-channel cancellation method that can reduce the dark straps. Moreover, Ma also generalizes the method to N-channel cancellation [31]. Another method is inverse power compensation [31], in which the amplitudes are compensated inverse to the powers, which will also mitigate the effect.

### 2.6. Complexity Analysis

To demonstrate the innovation more comprehensively, we discuss the complexities of our method and the classical Chirp Scaling (CS) algorithm and assume that the input data are $D\subset R^{N\times K}$. For fast Fourier transform (FFT) computing, we always extend the size to the nearest power of two: $D^{\prime }\subset R^{N_{2}\times K_{2}}$, $N_{2}=2^{m}\geq N$, $K_{2}=2^{n}\geq K$. The time cost of 128 FFT points and 128 dot product points on DSP are 4 μs and 2 μs, respectively. The major time cost of the CS algorithm is summarized in Table 1. From the table, we can obtain the total time cost of CS algorithm as

(9) $$T_{CS}=\frac{3KN_{2}+8K_{2}N_{2}}{256}$$

Similarly, we can summarize the major time cost of our method in Table 2. Additionally, the total time cost of our method is

(10) $$T_{our}=\frac{3NK_{2}}{256}$$

Comparing $T_{CS}$ and $T_{our}$, it is well-known that the complexity of our method is less than that of the CS algorithm. Our method just determines the FFT and the dot products once, and CS algorithm conducts them multiple times.

## 3. Experimental Studies

Nriet-SAR (SAR of Nanjing Research Institute of Electronic Technology), an airborne dual-band (L, X) multichannel polarimetric SAR sensor, was applied to conduct the experiment [33]. The principal parameters are shown in Table 3.

**Figure 11:**
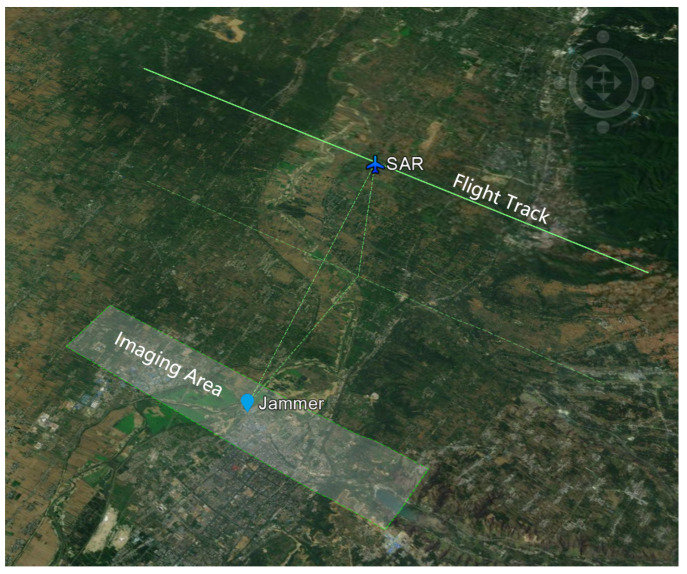
Geometric relationship of the SAR and the jammer.

The geometric relationship between the SAR sensor and the jammer is briefly illustrated in Figure 11, in which the radar moves along a linear flight path with a typical flight height of 7000 m, and the jammer is in the SAR imaging area.

**Figure 12:**
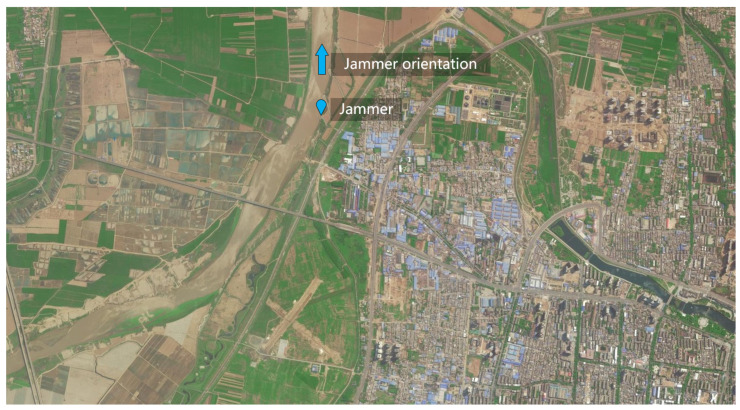
Optical image of the imaging area and the location and orientation of the jammer.

Figure 12 shows the optical image (from Google Earth) of the experimental area in Weinan City, Shanxi, China. The blue icon in the middle of Figure 12 indicates the location of the jammer (source of radiation) used in the experiment, and the blue arrow shows the antenna orientation of the transmitting and receiving antennas. Additionally, the flight direction of the Nriet-SAR platform is from south to north (from right to left in Figure 12).

**Figure 13:**
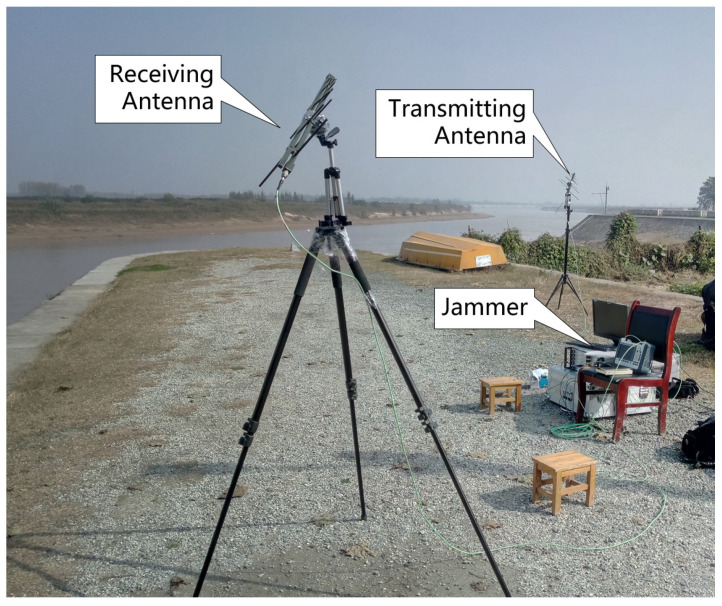
The jammer, the transmitting antenna, and the receiving antenna.

**Figure 14:**
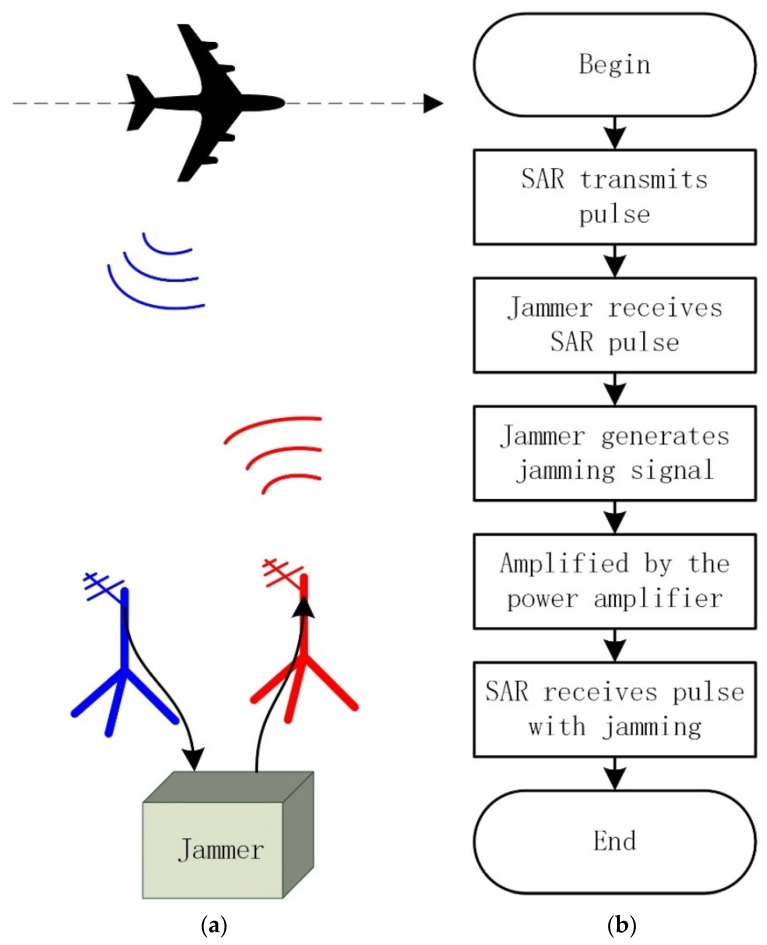
The schematic diagram and processing chain. (**a**) Schematic diagram. (**b**) Processing chain.

Figure 13 shows the experimental placement of the jammer, the transmitting antenna, and the receiving antenna. To clearly explain the working process of the jammer in detail, the schematic diagram and processing chain are shown in Figure 14. After the receiving antenna of the jammer receives the chirp signal transmitted from the radar, the jammer is triggered to generate the jamming signals. The jamming signals are transmitted by the transmitting antenna to the radar after being amplified by the power amplifier.

To verify the effectiveness of our proposed method, two linear SAR trajectories have been designed to observe the imaging area shown in Figure 12 in this experiment, in which the parameter configuration of SAR is shown in Table 1. In the first linear SAR trajectory, the jammer is turned off, which can be seen as a set of comparative experiments, so we can achieve a perfect SAR image of the imaging area with commonly used SAR imaging algorithms. In the other linear SAR trajectory, the jammer is turned on and transmits jamming signals to the receiving antenna of SAR according to the processing chain shown in Figure 14.

**Figure 15:**
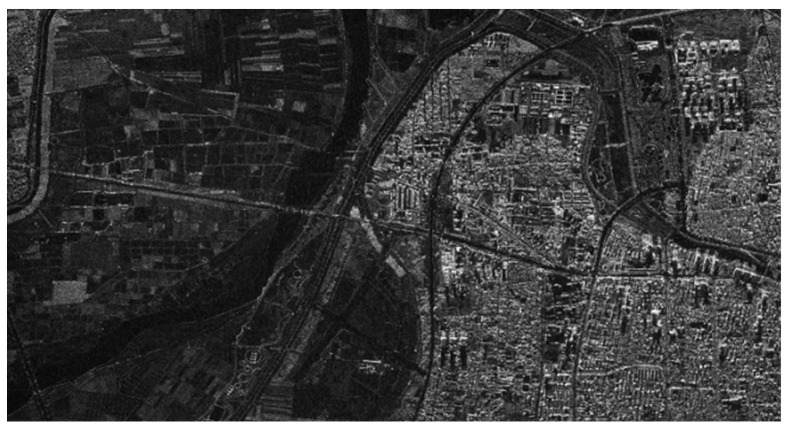
SAR image without jamming.

**Figure 16:**
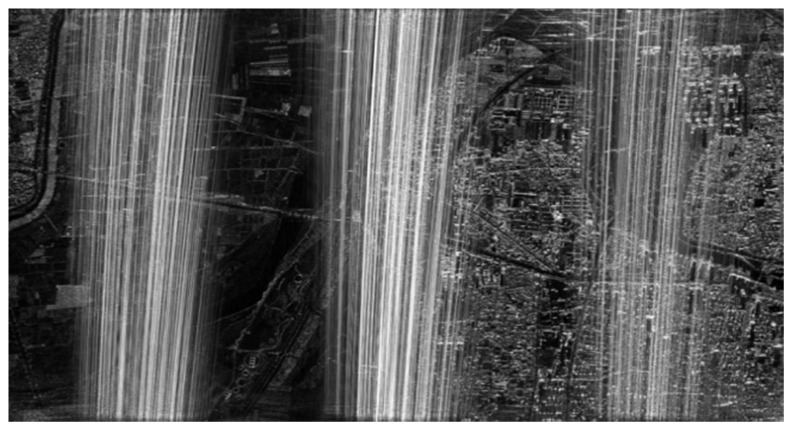
SAR image with jamming.

In this airborne experiment, the original SAR data obtained by Nriet-SAR are processed using along-track DCC. Figure 15 shows the SAR imaging result without jamming, and Figure 16 shows the SAR image with jamming. As we can see from Figure 15 and Figure 16, when the jammer is turned off, SAR can perfectly reconstruct the SAR image of the ground features in the imaging area. However, it can be clearly found that parts of the ground features are not clearly visible and that they are seriously influenced by the jamming signals in Figure 16.

**Figure 17:**
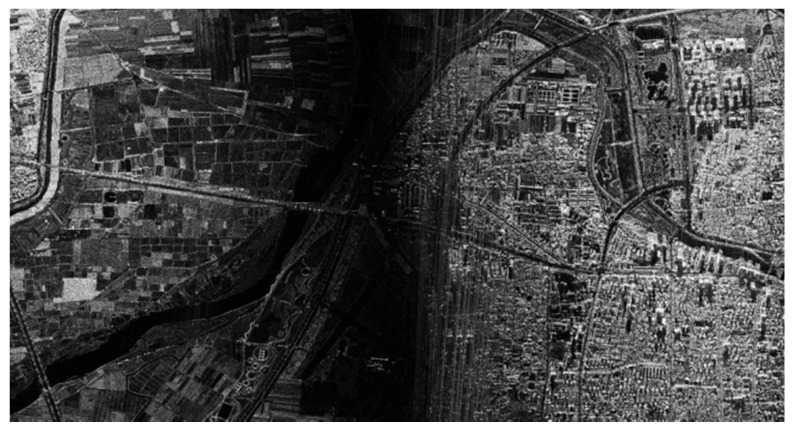
SAR image after DCC with relatively precise IPSR.

**Figure 18:**
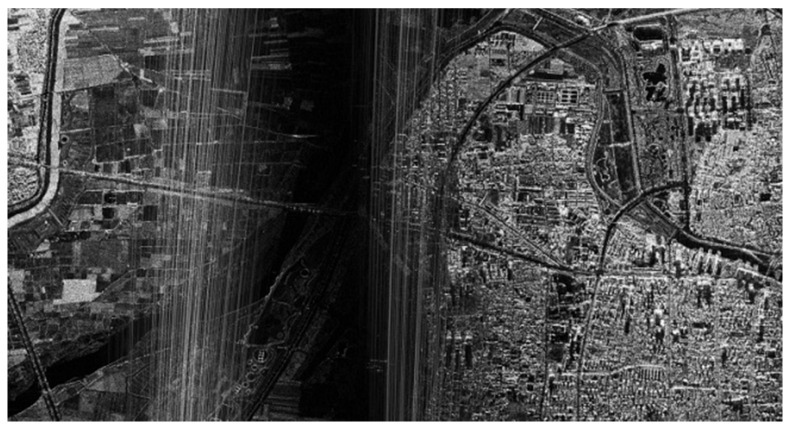
SAR image after DCC with IPSR error of 18°.

To eliminate the influence of the jamming signals and to reconstruct the SAR image of the imaging area, a cosine similarity-based DCC method has been applied in the airborne experimental data. Figure 17 and Figure 18 show the DCC images, while the IPSR error of Figure 18 is set to 18° on purpose, and the IPSR of Figure 17 is relatively precise.

As we can see from Figure 17, most of the interference images have been eliminated, and the ground features have become visible after accurate cosine similarity-based along-track DCC. Due to the set of the deliberate IPSR errors of 18°, which can be considered as the wrong estimations of the IPSR, Figure 18 suffers from the slight influence of the jamming signals. However, a dark strap still appears in the middle of Figure 17 and Figure 18 along the range direction of the jammer (from top to bottom), which is consistent with our analysis in Section 2.5.

Compared with the SAR image without jamming in Figure 15, the ground features in the imaging area can still be well reconstructed with our proposed method, while the SAR is severely influenced by the jammer.

To demonstrate the effectiveness of our method, the information entropy of image was used to reflect the potential of suppressing SAR jamming. Image entropy is a statistic based on information theory. The smaller the image entropy is, the more orderly the image is and the more information it contains. First, for a SAR image, we can calculate its entropy by

(11) $$H=-\overset{255}{\underset{i=0}{\sum }}p_{i}\log_{2}\left(p_{i}\right)$$

where $p_{i}$ is the probability of different grey values in the image. It was easy to calculate the entropy of the clear image, the jamming image, the image after DCC, and the image after DCC with an IPSR error of 18°: 4.8648, 5.1113, 4.9347, and 5.0248, respectively. The entropy of the image DCC is less than that of the jamming image, and it is greater than the entropy of the original image. Jamming would cause an increase in image entropy, and DCC could decrease the entropy of the jamming image. Overall, the results indicate that our method can suppress SAR jamming.

## 4. Discussion

In the practical application of along-track multichannel SAR, the channel error between channels mainly arises from the SAR system error, the baseline error, and the squint angle error. Related research illustrates that the channel error can influence the performance of channel cancellation operations. Specifically, multichannel SAR working at a higher frequency could be more sensitive to channel errors. In this situation, channel errors should be compensated for precisely before the multichannel SAR channel cancellation operation is performed.

This paper mainly focuses on the research on dual-channel SAR systems. As for N-channel SAR systems, the problem of dark straps can be perfectly solved after channel cancellation operations. In the N-channel SAR system, one channel can be chosen as the counter reference. Therefore, the channel cancellation operation of the N-channel SAR system can be regarded as a concatenation of multiple dual-channel SAR systems in which the proposed estimation method of IPSR based on cosine similarity can still be applied effectively.

In our future work, we will focus on the combination of channel error compensation and DCC operation for dual-channel SAR system. Moreover, the channel cancellation of the N-channel SAR system with much lower JSR will also represent the main direction of our future research.

## 5. Conclusions

In this paper, we study a dual-channel cancellation method for SAR jamming suppression that uses the interferometric phase of the jammer to eliminate the jamming signals via phase compensation and cancellation. To solve the problem of interferometric phase estimation under a low JSR, a novel method for IPSR estimation using cosine similarity is proposed, and several practical problems are discussed. An airborne experiment is conducted, and the preliminary results verify the effectiveness and practicality of the proposed method and show the great potential of the DCC method in SAR interference suppression.

## Figures and Tables

**Table 1:** The major time cost of the CS algorithm.

Operation	Input Data Size	Output Data Size	Time Cost (μs)
Azimuth FFT	N×K	$N_{2}\times K$	$K\times N_{2}/128\times 4$
Range K points dot product	$N_{2}\times K$	$N_{2}\times K$	$N_{2}\times K/128\times 2$
Range FFT	$N_{2}\times K$	$N_{2}\times K_{2}$	$N_{2}\times K_{2}/128\times 4$
Range $K_{2}$ points dot product	$N_{2}\times K_{2}$	$N_{2}\times K_{2}$	$N_{2}\times K_{2}/128\times 2$
Range IFFT	$N_{2}\times K_{2}$	$N_{2}\times K_{2}$	$N_{2}\times K_{2}/128\times 4$
Azimuth $N_{2}$ points dot product	$N_{2}\times K_{2}$	$N_{2}\times K_{2}$	$N_{2}\times K_{2}/128\times 2$
Azimuth IFFT	$N_{2}\times K_{2}$	$N_{2}\times K_{2}$	$N_{2}\times K_{2}/128\times 4$

**Table 2:** The major time cost of our method.

Operation	Input Data Size	Output Data Size	Time Cost (μs)
N times FFT	N×K	$N\times K_{2}$	$N\times K_{2}/128\times 4$
N times $K_{2}$ points dot product	$N\times K_{2}$	$N\times K_{2}$	$N\times K_{2}/128\times 2$

**Table 3:** Main parameters of Nriet-SAR L-band sensor.

Parameter	Value
Frequency band	L
Bandwidth	100 MHz
Pulse repeat time (PRF)	800 Hz
Noise equivalent sigma zero (NESZ)	<−24 dB
Range resolution	1.5 m
Azimuth resolution	0.6 m
Channel number	2
Channel distance	0.6 m
Typical flight height	7000 m

## Data Availability

The data that support the findings of this study are available upon request from the corresponding author. The data are not publicly available due to privacy restrictions.

## Abbreviations

Abbreviation	Full Name
HRWS	High-resolution and wide-swath
DCC	Dual-channel cancellation
JSR	Jamming-to-signal-ratio
SAR	Synthetic aperture radar
InSAR	Interferometric SAR
IPSR	Interferometric phase of the source of radiation
IPDC	Interferometric phase of the dual channels
PRT	Pulse repeat time
CS	Chirp Scaling
FFT	Fast Fourier transform
IFFT	Inverse fast Fourier transform
PRF	Pulse repeat frequency
NESZ	Noise equivalent sigma zero
